# Two genetic variants in the *SRD5A2* gene are found to be associated with sex differences in the disease characteristics of patients with chronic hepatitis B virus infection

**DOI:** 10.1186/s13293-023-00553-4

**Published:** 2023-10-03

**Authors:** Honglei Duan, Xu Wang, Wenqian Qi, Jingyi Shi, Liang Han, Guohua Wang, Yanhui Xu, Jia Liu, Jiangbin Wang

**Affiliations:** 1 Department of Gastroenterology, China-Japan Union Hospital of Jilin University, No. 126 Xiantai St., Changchun, 130033 Jilin China; 2 Department of Pathology, China-Japan Union Hospital of Jilin University, Changchun, 130033 Jilin China; 3 Department of Gastroenterology and Nephrology, Songyuan Jilin Oilfield Hospital, Songyuan, 138000 Jilin China; 4 Department of Gastroenterology and Hepatology, People’s Hospital of Zhengzhou University and Henan Provincial People’s Hospital, Zhengzhou, 450003 Henan China

**Keywords:** Hepatitis B virus, Chronic HBV infection, SRD5A2, Single nucleotide polymorphism, Sex hormone, Sex hormone receptor, Sex difference

## Abstract

**Background:**

To examine the expression characteristics of single nucleotide polymorphisms (SNPs) in the *SRD5A2* gene and investigate their potential association with differences in the clinical characteristics between sexes in patients with chronic hepatitis B virus (HBV) infection.

**Methods:**

A total of 30 loci in six genes primarily involved in the metabolism and signaling of sex hormones/sex hormone receptors, namely AKR1C2, AKR1C3, HSD17B6, SRD5A1, SRD5A2, and ESR1, were genotyped in 1007 patients from eight counties (cities) in Northeastern China with chronic HBV infection and 1040 healthy controls, and their association with viral replication characteristics and the differences in disease severity between sexes was assessed. Western blotting was conducted to determine the hepatic SRD5A2 protein level and its relationship with the inflammatory activity and fibrosis degree in male and female patients.

**Results:**

Two SNP loci in the *SRD5A2* gene (rs12470143 and rs7594951) exhibited significant differences in genotype and allele frequencies between sexes, with the proportion of T alleles significantly higher in males than in females. It was found that the incidence and severity of HBV-related liver fibrosis were significantly higher in patients with the T/T genotype in SRD5A2 rs12470143 and rs7594951 than those with the non-T/T genotype. Additionally, serum HBV DNA levels were significantly elevated in T/T patients compared to non-T/T patients. Female patients exhibited significantly lower serum DNA levels compared to male patients. Western blot analysis indicated that greater hepatic SRD5A2 protein levels were associated with higher METAVIR inflammation and fibrosis scores. Furthermore, multivariate analysis showed that the two genetic variants in the SRD5A2 gene (rs12470143 C > T, r7594951 C > T), together with the male sex, age > 50 years old, HBeAg positive status, elevated serum HBsAg load, high serum HBV DNA load, and HBV genotype C, were independent risk factors for HBV-related liver fibrosis.

**Conclusions:**

This study demonstrated that two genetic variants in the *SRD5A2* gene (rs12470143 C > T, r7594951 C > T) are associated with sex differences in the clinical characteristics of patients with chronic HBV infection.

**Supplementary Information:**

The online version contains supplementary material available at 10.1186/s13293-023-00553-4.

## Highlights

- There are significant differences in genotype and allele frequencies between male and female patients with chronic hepatitis B virus (HBV) infection at two single nucleotide polymorphism loci (rs12470143 and rs7594951) in the *SRD5A2* gene.
- The proportion of T alleles at the rs12470143 and rs7594951 loci of the *SRD5A2* gene are more prevalent in male patients than in female patients.
- T/T genotypes at the rs12470143 and rs7594951 loci of the *SRD5A2* gene exhibit a significant association with a higher prevalence and severity of HBV-related liver fibrosis, as well as higher levels of HBV DNA, in patients with chronic HBV infection.
- Elevated hepatic SRD5A2 protein levels in patients with chronic HBV infection are significantly associated with more severe liver inflammation and fibrosis.

**Supplementary Information:**

The online version contains supplementary material available at 10.1186/s13293-023-00553-4.

## Plain language summary

This study genotyped 30 genetic loci in six genes primarily involved in the metabolism and signaling pathways of sex hormones/sex hormone receptors, including AKR1C2, AKR1C3, HSD17B6, SRD5A1, SRD5A2, and ESR1, in 1007 patients with chronic hepatitis B virus (HBV) infection and 1040 healthy controls. It was found that two single nucleotide polymorphism (SNP) loci in the *SRD5A2* gene (rs12470143 and rs7594951) showed significant differences in genotype and allele frequencies between male and female patients. Further, the proportion of the T alleles was significantly higher in males than in females. The study also found that patients with the T/T genotype had a higher incidence and severity of HBV-related liver fibrosis compared to those with other genotypes. Additionally, serum HBV DNA levels were significantly higher in T/T patients compared to non-T/T patients. Female patients had lower serum DNA levels compared to male patients. Further analysis showed that higher levels of the SRD5A2 protein were associated with increased inflammation and fibrosis scores in the liver. Multivariate analysis revealed that the two genetic variants in the SRD5A2 gene, together with male sex, age over 50, HBeAg positive status, elevated serum HBsAg load, high serum HBV DNA load, and HBV genotype C, were independent risk factors for HBV-related liver fibrosis. In summary, this study demonstrated that genetic variations in the SRD5A2 gene are associated with differences in the clinical characteristics of male and female patients with chronic HBV infection.

**Supplementary Information:**

The online version contains supplementary material available at 10.1186/s13293-023-00553-4.

## Background

Sex disparity in disease regression is a notable phenomenon observed in patients with chronic hepatitis B virus (HBV) infection; whereby, male patients with HBV infection, compared to female patients, exhibit several distinct characteristics, including higher levels of serum HBsAg, increased likelihood of chronicity, rapid disease progression, higher incidence of cirrhosis and even liver cancer, and a relatively poor prognosis [1]. The male-to-female ratio among asymptomatic carriers of HBV is 1.2, which increases to 6.3 during the development of chronic hepatitis B (CHB), and is further elevated to 9.8 in cases of HBV-associated hepatocellular carcinoma (HCC) [2]. These significant sex differences in the incidence of CHB and related HCC have prompted extensive research on the therapeutic effects of sex hormone intervention. However, attempts to alter the prognosis of HBV-infected experimental animals through simple sex intervention or exogenous hormone supplementation have been unsuccessful, suggesting that the involvement of sex hormone/sex hormone receptor-mediated signaling pathways and downstream molecules at specific regulatory points may contribute to inter-sex differences following HBV infection.

Hepatocytes express both androgen receptors (ARs) and estrogen receptors, but their impact on HBV mRNA transcriptional activity through the respective estrogen and androgen signaling pathways differs considerably. HBV nucleotide sequences in the 900–1350 region, known as the HBV enhancer I region, play a crucial role in regulating viral transcription. Within this region, there are two specific androgen response element (ARE) regions, located in the 913–927 and 949–963 regions, respectively. The interaction between androgen and ARs on the surface of hepatocytes leads to the binding of the ARs to the viral ARE, which in turn promotes the transcription of HBV mRNA and the development and progression of the disease. In contrast, the effects of estrogen on HBV transcription differ largely from those of androgen. Estrogen exerts its impact through two estrogen receptors: ESR-1 and ESR-2. For instance, when estrogen binds to ESR-1 on the surface of hepatocytes, it physically interacts with hepatocyte nuclear factor 4α (HNF4α), which prevents the binding of HNF4α to viral enhancer I, resulting in a decreased activity of viral enhancer I, and the inhibition of viral mRNA transcription [3]. This might explain why female patients with chronic HBV infection tend to have prolonged periods of low HBV replication and may even remain in a more stable disease state for an extended duration.

Aldosterone reductase 1C2 (AKR1C2), aldosterone reductase 1C3 (AKR1C3), 17β-hydroxysteroid dehydrogenase 6 (HSD17B6), steroid 5-alpha-reductase type 1 (SRD5A1), steroid 5-alpha-reductase type 2 (SRD5A2), and estrogen receptor 1 (ESR1) are key enzymes in the synthesis and functioning of sex hormones. AKR1C2 [4], an important enzyme in the androgen metabolism pathway, converts dihydrotestosterone to 3-alpha-diol. AKR1C3 [5] reduces low biologically active androstenedione to testosterone or dehydroepiandrosterone to androstenediol, which in turn is converted to testosterone. HSD17B6 [6] possesses redox properties that convert 3-α diol to dihydrotestosterone; SRD5A1 and SRD5A2 [7], two 5α reductases in the androgen synthesis pathway, primarily convert testosterone to the more biologically active dihydrotestosterone; ESR1 [3] binds to estrogen and physically inhibits the binding of HNF4α to viral enhancer I, which subsequently hinders HBV mRNA transcription. Mutations in the aforementioned genes involved in the sex hormone/sex hormone receptor signaling pathway may affect the sex hormone receptor binding and thus the progression of sex hormone-related diseases. In the context of chronic HBV infection, the mutations can also alter HBV mRNA transcription, leading to differences in disease regression between sexes (Additional file 1: Fig. S1).

The differences in disease regression between sexes in chronic hepatitis C virus (HCV) infection are not as pronounced as those observed in chronic HBV infection. In a United States (US) epidemiological survey study, HCV infection was found to be 1.99–2.57 times more prevalent in men compared to women, and men were over three times more likely to develop HCV-associated HCC than women [8–11]. Some studies [12] have shown that estrogen (E2) can interfere with the late phase of the HCV life cycle, specifically the assembly/release process via binding to intracellular receptors and inhibiting HCV replication. Furthermore, E2 has been shown to impede HCV entry into hepatocytes and the subsequent replication processes through binding to G protein-coupled receptor 30, eventually leading to the inhibition of viral replication (Additional file 1: Fig. S2) [13]. An Italian analysis of single nucleotide polymorphisms (SNPs) in genes (CYP17A1, SRD5A2, and COMT) involved in the sex hormone/sex hormone receptor signaling pathway examined 387 patients with chronic HCV infection (22% of whom were women) [14]. Compared to the asymptomatic carriers, the CYP17(-34)C/C genotype showed a higher expression in hepatocellular carcinoma (HCC) patients (22.5 vs. 11.2%, *p* = 0.05). Among the female patients, the postmenopausal women had a significantly higher risk of concurrent HCC (*p* = 0.03), suggesting that a functional SNP in the CYP17A1 gene increases the risk of liver disease progression in postmenopausal women. In a study conducted in the US [15], 16 candidate genes (AR, AKR1, AKR1C2, AKR1C3, CYP17A1, CYP19A1, ESR1, ESR2, GPER, HSD17B6, HSD3B1, HSD3B2, SHBG, SRD5A1, SRD5A2, UGT2B17) in the sex hormone/sex hormone receptor signaling pathway were analyzed, including up to 472 SNPs. The results indicated that four regulatory genes (AKR1C2, AKR1C3, HSD17B6, ESR1) had 9 SNPs closely associated with sex hormone synthesis and metabolism, which were identified as risk factors for progressive liver fibrosis in chronic HCV infection, while 4 SNPs in three genes (AKR1C2, AKR1C3, SRD5A1) were risk factors for progressive liver inflammation.

Although chronic HBV infection displays a more notable disparity in disease regression between sexes and a more robust response in the sex hormone/sex hormone receptor signaling pathway compared to chronic HCV infection, our knowledge of the genetic factors associated with chronic HBV and related liver diseases is relatively limited in comparison to HCV infection. Therefore, we conducted this study to analyze 30 loci in 6 important genes involved in estrogen/androgen metabolism and the signaling pathways. The specific aims of this study were to investigate their relationship with disease progression and identify the genetic variations associated with sex differences in patients with chronic HBV infection.

## Methods

### Study subjects

In this study, a total of 1007 patients with chronic HBV infection were retrospectively recruited from eight counties/cities in Northeastern China, namely Changchun, Jilin, Yanji, Songyuan, Siping, Tonghua, Baishan, and Baicheng [16]. The chronic HBV-infected patients met the diagnostic criteria for chronic HBV infection according to clinical guidelines developed by the Chinese Medical Association (Guidelines for the prevention and treatment of chronic hepatitis B, 2019) [17]. The inclusion criteria for patients with chronic HBV infection were as follows: (1) age ≥ 18 years old; (2) serum HBsAg positive status for more than 6 months and serum HBV DNA quantitative positive status; (3) not receiving antiviral therapy at the time of enrollment. Of these patients, 951 were treatment-naïve patients with complete personal data and peripheral anticoagulated whole blood specimens who had not undergone liver biopsy, while 56 underwent percutaneous liver biopsy for a definitive diagnosis. The liver biopsies of these patients were obtained from the tissue specimen bank of China-Japan Union Hospital of Jilin University.

In addition, 1040 age-matched healthy individuals with a negative status of serum HBsAg and HBV DNA were included as controls. The following inclusion criteria were used for the healthy controls: (1) age ≥ 18 years old; (2) serum HBsAg negative status; (3) serum HBV DNA negative status.

The exclusion criteria were as follows: (1) chronic HCV infection combined with autoimmune liver disease, alcoholic liver disease, hepatic schistosomiasis, drug-related liver disease, genetic metabolic liver disease, or other liver diseases; (2) chronic HCV infection combined with liver cancer or other types of tumors; (3) recent use of steroid drugs and chronic HCV infection combined with pituitary tumors, polycystic ovary syndrome, or other diseases affecting hormone synthesis and secretion.

All the study subjects provided informed consent. This study was approved by the Ethics Committee of China-Japan Union Hospital of Jilin University (No. 2016- nsfc015) and was performed in compliance with the Declaration of Helsinki.

### Clinical characteristics, laboratory tests, and virological characteristics

All the included patients were analyzed for determining their clinical characteristics, serum biochemical parameters, and serum virological parameters (i.e., HBV DNA quantification, hepatitis B serum markers, HBV genotype). Liver stiffness measurements (LSMs) were performed using transient ultrasound elastography (Fibroscan, Echosens, France) and expressed as E (kPa). The liver stiffness E values were graded according to the World Federation for Ultrasound in Medicine and Biology (WFUMB) 2015 guidelines [18]: no or mild fibrosis (corresponding to METAVIR stage F0–F1), E = 2.5–7.0 kPa; significant fibrosis (corresponding to METAVIR stage F2), E = 7.1–9.5 kPa; severe fibrosis (corresponding to METAVIR stage F3), E = 9.6–12.5 kPa; and cirrhosis (corresponding to METAVIR stage F4), E > 12.5 kPa.

Based on the approximate trend of sex hormone levels during reproductive aging, female patients were categorized into two groups: age ≤ 50 years old (i.e., reproductive women) and age > 50 years old (i.e., late menopausal women). Similarly, male patients were divided into two groups: age ≤ 50 years old and age > 50 years old [19, 20].

### Single nucleotide polymorphism (SNP) analysis

The DNA extraction of blood samples was performed using the Blood Genomic DNA Rapid Extraction Kit (Shanghai Bioengineering Co., Shanghai, China). Primer Premier 5.0 software was utilized for designing SNP primers associated with AKR1C2, AKR1C3, HSD17B6, SRD5A1, SRD5A2, and ESR1. These primers were synthesized by Shanghai Bioengineering Co. Amplification of the SNP site sequences and the preparation of compatible Illumina sequencing libraries were accomplished through a two-step polymerase chain reaction (PCR). The resulting PCR products were then purified and recovered using AMPure XP magnetic beads. Equal amounts of individual PCR products were combined and sequenced using a HiSeq XTen sequencer (Illumina, San Diego, CA, USA). The key genes and primers are listed below.

*AKR1C3* gene:

Forward primer: 5ʹ-CCCAGGTTCAATAGGAAAGAA-3ʹ, reverse primer: 5ʹ-ACCTTCACCCATGCACTTTC-3ʹ;

SNPs: rs2211623, rs2186174, rs2398203, rs2154306, rs4559587, rs12529, rs3763676, rs2096421.

*AKR1C2* gene:

Forward primer: 5ʹ-CCGTCAAATTGGCAATAGAAGCC-3ʹ, reverse primer: 5ʹ-CAACTCTGGTCGATGGGAATTGCT-3ʹ;

SNPs: rs2801904, rs12414884.

*HSD17B6* gene:

Forward primer: 5ʹ-TCTTTGTGGGAGAGTAGCTTC-3ʹ, reverse primer: 5ʹ-TTTGTCAGTCTCTTCCTTTCTCATC-3ʹ;

SNPs: rs4237805, rs898611.

*SRD5A1* gene:

Forward primer: 5ʹ-GGTTTTGGCTTGTGGTTAACA-3ʹ, reverse primer: 5ʹ-CTCTTCAAATTTCCGGAGGTAC-3ʹ;

SNPs: rs248799, rs248800, rs6872996, rs3797177, rs1691053.

*SRD5A2* gene:

Forward primer: 5ʹ-CGCGAAGTGATCCAGAAAC-3ʹ, reverse primer: 5ʹ-CAGGACCAGGTAGCCTGTG-3ʹ;

SNPs: rs2208532, rs12470143, rs523349, rs4952197, rs7594951, rs612224.

*ESR1* gene:

Forward primer: 5ʹ-CUGUCUUCUGUUGUGGGAACA-3ʹ, reverse primer: 5ʹ-GGAGAAUGUUGAAACACAAUU-3ʹ;

SNPs: rs6909023, rs6920483, rs2077647, rs2234693, rs1062577, rs7753153, rs9340799.

### Measurement of the METAVIR inflammatory fibrosis score and SRD5A2 protein expression level in liver tissue

Liver tissues from all the patients were fixed with formaldehyde, followed by routine dehydration and embedding in paraffin. Subsequently, the tissues were stained with hematoxylin–eosin (H&E) for pathological examination. The histopathological assessment of liver inflammation and fibrosis in chronic HBV-infected patients was conducted using the METAVIR scoring system as reported previously [21]. In this system, the inflammatory activity score A ranges from 0 to 3, while the fibrosis score (F) ranges from 0 to 4.

The primary antibody used in this study was SRD5A2 antibody (Anti-SRD5A2 antibody [EPR6281(B)]) obtained from Shanghai Uninvest Biotechnology Co. (Shanghai, China). The secondary antibody used was Goat-Anti-Rabbit IgG-HRP, diluted at a ratio of 1:10,000. Protein lysates were prepared from liver tissue using the Protein Extraction Kit (C510003-0050, Biotech), with the addition of broad-spectrum protease inhibitors and phosphatase inhibitors to the lysate. The protein concentration was determined using the BCA Protein Assay Kit (C503021-0500, Biotech). Subsequently, 20 μg of protein was mixed with the loading buffer and loaded into a 10–15% SDS–polyacrylamide gel electrophoresis system. The separated proteins were then electrotransferred onto a PVDF membrane. The resulting membrane was then incubated overnight at 4 ℃ with the primary antibody, followed by washing at room temperature for 2 h with the secondary antibody. Also, β-actin was used as an internal reference control to normalize the protein expression. The gray value of the bands was determined using Image J software, with the quantified value representing the ratio of the target protein to the internal reference protein.

### Statistical analysis methods

Genetic equilibrium was assessed using the Hardy–Weinberg Equilibrium test (HWE) to determine the genotype frequencies, while the allele and genotype frequencies between groups were compared using the chi-square test. Count data were presented as frequencies and percentages, and statistical analysis was performed using either the chi-square test or Fisher's exact probability method. Measurement data were described by the mean and standard deviation (SD), and statistical analysis was performed using the t-test. Factor screening was carried out using logistic regression analysis, followed by sequential univariate and multivariate analyses. Variable screening was performed using the stepwise regression method. Differences were considered statistically significant at *p* < 0.05. Data analysis and graphical representation were conducted using SPSS 22.0 and GraphPad Prism 8 software.

## Results

### Demographic and clinical characteristics of the patients with chronic HBV infection and the control individuals

The demographic and clinical characteristics of the studied patients with chronic HBV and the control individuals are summarized in Table 1. Comparative analysis showed that there were no significant differences in the mean age, history of smoking, history of alcohol consumption, history of hypertension, history of fatty liver disease, BMI, or HOMA-IR between the two groups (Table 1). However, the proportion of males, and the AST and ALT levels were significantly higher in the chronic HBV group compared to the healthy controls. Additionally, the albumin levels, cholinesterase, and platelet count were significantly lower in the chronic HBV group compared to the healthy controls. In the chronic HBV group, the HBeAg positivity rate was 82.1%; median HBsAg quantification was 3.75 log10IU/ml; median serum HBV DNA quantification was 6.3 log10IU/ml; the HBV virus genotype was type B in 206 cases (20.5%) and type C in 801 cases (79.5%); and the median liver fibrosis F0–F2 stage liver hardness value (kPa) was 7.6, and 11.3 for stage F3–F4, as detailed in Table 1.

**Table 1:** Demographic and clinicopathological characteristics of the patients with chronic HBV and the controls

Characteristics	Chronic HBV patients (n = 1007)	Controls (n = 1040)	P-value
Age, years	48.19 ± 11.3	49.31 ± 10.57	0.058
Male/female, %	65.9/34.1	61.6/38.4	**0.024**
Smoking, n (%)	224 (22.3%)	198 (19.3%)	0.080
Drinking, n (%)	291 (28.9%)	265 (25.5%)	0.091
Hypertension*, n (%)	166 (16.5%)	147 (14.1%)	0.140
Fatty liver, n (%)	194 (19.3%)	225 (21.6%)	0.184
BMI, kg/m^2^	24.11 (22.49, 26.70)	25.04 (22.20, 27.91)	0.118
HOMA-IR	2.33 (1.96, 2.66)	2.36 (1.96, 2.68)	0.766
ALT, IU/L	59.66 (32.97, 87.08)	27.19 (19.67, 39.39)	** < 0.001**
AST, IU/L	57.60 (33.00, 86.90)	26.73 (22.03, 34.82)	** < 0.001**
ALB, g/L	31.70 (28.70, 35.30)	37.05 (31.02, 41.81)	** < 0.001**
Cholinesterase, IU/L	9080 (7620, 10,460)	10,904 (9001, 13,406)	** < 0.001**
PLT (10^9^/L)	101 (81, 122)	186 (117.25, 250)	** < 0.001**
HBeAg positive, %	827 (82.1%)		
HBsAg (log10IU/ml))	3.75 (3.55, 3.89)		
HBV DNA (log10IU/ml)	6.3 (5.14, 7.7)		
HBV genotype 2/3, %	20.5/79.5		
E, kPa			
F0–2	7.6 (6.75, 8.4)		
F3–4	11.3 (10.1, 12.6)		

Count data: n (%) indicated; measurement data conform to a normal distribution: mean ± standard deviation; measurement data do not follow a normal distribution: median (interquartile spacing). *Here and below, hypertension was defined as systolic blood pressure > 140 mmHg or diastolic blood pressure > 90 mmHg, *BMI* body mass index, *HOMA-IR* insulin resistance index, *ALT* glutamic aminotransferase, *AST* glutamic aminotransferase

### Identification of the SNP loci associated with chronic HBV infection, and comparison of the genotype and allele frequencies between male and female patients

Genotyping and analysis of 30 loci in 6 genes (AKR1C2, AKR1C3, HSD17B6, SRD5A1, SRD5A2, and ESR1) revealed 23 SNP loci, including 2 SNP loci in the AKR1C2 gene, 3 in the AKR1C3 gene, 2 in the HSD17B6 gene, 3 in the SRD5A1 gene, 6 in the SRD5A2 gene, and 7 in the ESR1 gene (Table 2). Comparison of the frequencies of the 23 SNP loci indicated that there was no difference between the chronic HBV-infected patients and healthy controls (Table 2).

**Table 2:** Single nucleotide polymorphism analysis of 30 loci in 6 genes in chronic HBV-infected patients and healthy controls

Gene	dbSNPrs#	Position	dbSNPallele	Type	Chronic HBV-infected patients gene frequency (n = 1007)	Healthy controls gene frequency (n = 1040)	P value
AKR1C2	rs2801904	chr10:5041692	C > A	Intron	0.757	0.750	0.758
	rs12414884	chr10:5021206	T > G	Intron	0.189	0.136	0.200
AKR1C3	rs2096421	chr10:5055213	G > A	Intron	0.750	0.735	0.449
	rs2398203	chr10:5134591	C > T	Intron	0.189	0.184	0.777
	rs2186174	chr10:5134535	A > C	Intron	0.189	0.188	0.955
	rs2211623	chr10:5062949	G > C	Intron	0	0	–
	rs2154306	chr10:5101710	T > C	Intron	0	0	–
	rs4559587	chr10:5100663	C > A	Intron	0	0	–
	rs12529	chr10:5094459	C > G	Missense	0	0	–
	rs3763676	chr10:5094307	A > G	Intron	0	0	–
HSD17B6	rs4237805	chr12:56855861	G > A	Intron	0.156	0.140	0.350
	rs898611	chr12:56769532	C > A	Intron	0.802	0.799	0.868
SRD5A1	rs3797177	chr5:6666971	T > A	Intron	0.216	0.211	0.747
	rs248800	chr5:6660287	C > A	Intron	0.223	0.207	0.361
	rs1691053	chr5:6677052	T > C	Intron	0.220	0.027	0.417
	rs248799	chr5:6694579	G > A	Intron	0	0	–
	rs6872996	chr5:6661853	C > T	Intron	0	0	–
SRD5A2	rs2208532	chr2:31563919	G > A	Intron	0.405	0.384	0.320
	rs12470143	chr2:31538488	C > T	Intron	0.198	0.286	0.813
	rs523349	chr2:31580636	G > C	Missense	0.422	0.418	0.893
	rs4952197	chr2:31542061	A > G	Intron	0.564	0.544	0.374
	rs7594951	chr2:31566723	C > T	Intron	0.103	0.080	0.066
	rs612224	chr2:35186801	C > A	Intron	0.051	0.049	0.452
ESR1	rs6909023	chr6:151832562	G > A	Intron	0.137	0.096	0.974
	rs6920483	chr6:151810186	G > A	Intron	0.117	0.114	0.890
	rs2077647	chr6:151807942	T > C	Exonic	0.367	0.257	0.493
	rs2234693	chr6:151842200	T > C	Intron	0.385	0.368	0.438
	rs1062577	chr6:152102770	T > A	UTR3	0.310	0.306	0.848
	rs7753153	chr6:151826963	G > A	Intron	0.243	0.238	0.836
	rs9340799	chr6:151842246	A > G	Intron	0.204	0.198	0.783

Furthermore, we compared both the genotype and allele frequencies of the 23 SNP loci in 6 genes (AKR1C2, AKR1C3, HSD17B6, SRD5A1, SRD5A2, and ESR1) between male and female patients with chronic HBV. Only the two SNP loci in the SRD5A2 gene (rs12470143 and rs7594951) showed significant differences in the genotype and allele frequencies between the male and female patients (all *p* < 0.05) (Table 3), whereas the 21 SNPs loci in the remaining genes exhibited no differences (Additional file 1: Table S1). Among the two loci that differed by sex, the frequency of T allele expression at rs 12,470,143 and rs 7,594,951 of the *SRD5A2* gene was significantly higher in men than in women, with *p* < 0.05 (Table 3).

**Table 3:** Genotype and allele frequencies of two genetic variants in the *SRD5A2* gene between male and female patients with chronic HBV infection

Variants	Male, n (%)	Female, n (%)	*χ*^*2*^	P value
*SRD5A2* rs12470143 Genotype				
CC	461 (69.4%)	294 (85.7%)	44.949	** < 0.001**
CT	161 (24.2%)	24 (7.0%)		
TT	42 (6.3%)	25 (7.3%)		
Allele				
C	1083 (81.6%)	611 (89.1%)	19.119	** < 0.001**
T	245 (18.4%)	75 (10.9%)		
*SRD5A2* rs7594951 Genotype				
CC	538 (81.0%)	309 (85.7%)	33.186	** < 0.001**
CT	90 (13.6%)	8 (6.7%)		
TT	36 (5.4%)	26 (7.6%)		
Allele				
C	1166 (87.8%)	626 (91.3%)	5.497	**0.019**
T	162 (12.2%)	60 (8.7%)		

### Association of SRD5A2 rs12470143 and rs7594951 with the prevalence and severity of liver fibrosis, and differences between male and female patients

**Fig. 1:**
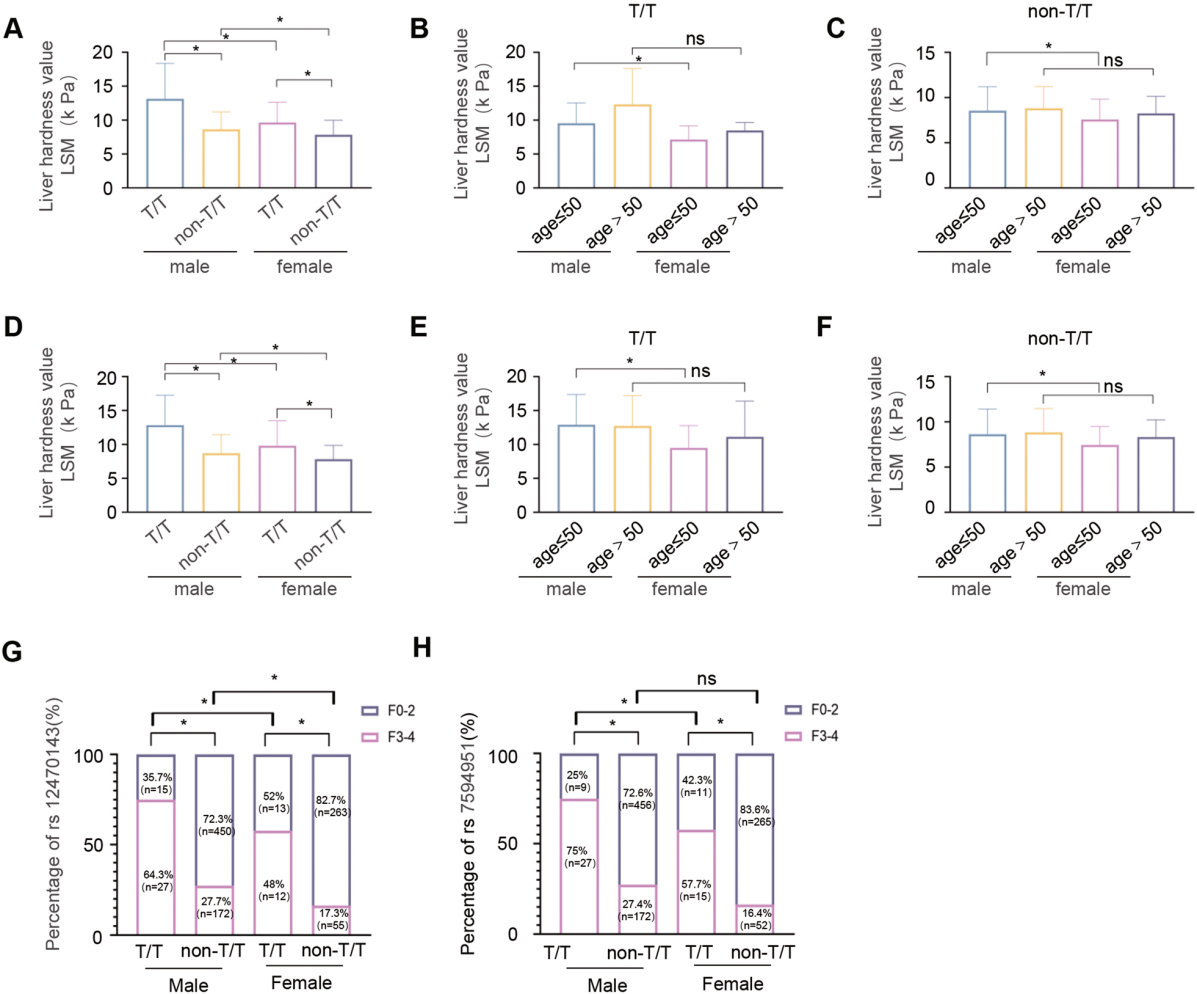
Association of SRD5A2 rs12470143 and rs7594951 with the severity of liver fibrosis and the differences among different age and sex groups. Comparison of liver stiffness (LSM value) among **A** male and female patients with the T/T or non-T/T genotype, **B** male and female patients stratified by age with the T/T genotype, **C** male and female patients stratified by age with the non-T/T genotype at the rs12470143 locus of the *SRD5A2* gene; Comparison of liver stiffness (LSM value) among **D** male and female patients with the T/T or non-T/T genotype, **E** male and female patients stratified by age with the T/T genotype, **F** male and female patients stratified by age with the non-T/T genotype at the rs7594951 locus of the *SRD5A2* gene. ns, *p* > 0.05; *, *p* < 0.05; **G** Comparison of the degree of liver fibrosis between patients with different sexes and genotypes at the rs12470143 locus of the *SRD5A2* gene; **H** Comparison of the degree of liver fibrosis between patients with different sexes and genotypes at the rs7594951 locus of the *SRD5A2* gene

Liver advanced fibrosis was found to be significantly more prevalent and severe in patients with the T/T genotype at the rs12470143 and rs7594951 loci of the *SRD5A2* gene compared to those with the non-T/T genotype (Table 4). Further stratification of the patients by sex and age revealed that liver fibrosis was significantly more severe in male patients than in females, regardless of the T/T or non-T/T genotype (*p* < 0.05). Among the patients aged ≤ 50 years old, the liver stiffness values were higher in men than in women, regardless of the T/T or non-T/T genotype. However, among the patients aged > 50 years old, there was no significant difference in the liver stiffness values in women compared with men of the same age, regardless of the T/T or non-T/T genotype (Fig. 1A–F). Furthermore, the severity of liver fibrosis at the rs12470143 and rs7594951 loci of the SRD5A2 gene was compared between patients of different sexes and ages, specifically between those with the T/T genotype and those with the non-T/T genotype. As shown in Figs. 1G and H, patients with the T/T genotype and the same sex and loci exhibited more severe liver fibrosis compared to those with the non-T/T genotype.

**Table 4:** Comparison of the genotype and allele frequencies of *SRD5A2* rs12470143 and rs7594951 between different liver fibrosis stages in patients with chronic HBV infection

Variant	F0–2, n (%)	F3–4, n (%)	*χ*^*2*^	P value
*SRD5A2* rs12470143 Genotype				
CC	560 (75.6%)	165 (62.0%)	40.479	** < 0.001**
CT	153 (20.6%)	62 (23.3%)		
TT	28 (3.8%)	39 (14.7%)		
Allele				
C	1273 (85.9%)	392 (73.7%)	78.601	** < 0.001**
T	179 (14.1%)	140 (26.3%)		
*SRD5A2* rs7594951 Genotype				
CC	648 (87.4%)	199 (74.8%)	58.253	** < 0.001**
CT	73 (9.9%)	25 (9.4%)		
TT	20 (2.7%)	42 (15.8%)		
Allele				
C	1369 (92.4%)	423 (79.5%)	66.050	** < 0.001**
T	113 (7.6%)	109 (20.5%)

### Association of SRD5A2 rs12470143 and rs7594951 with serum HBV DNA levels and differences among the different sexes and age groups

**Fig. 2:**
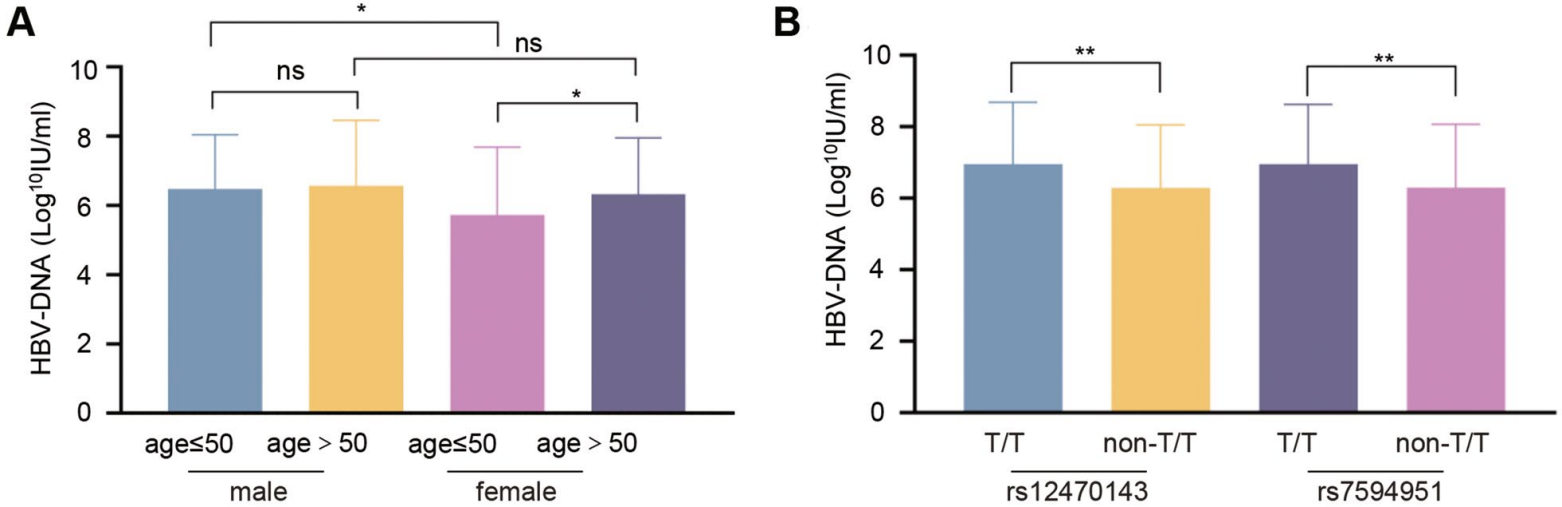
Association of SRD5A2 rs12470143 and rs7594951 with serum HBV DNA levels and differences among different age and sex groups. **A** Comparison of HBV DNA levels among different age and sex groups; **B** Comparison of HBV DNA levels among patients with the T/T or non-T/T genotype at the rs12470143 and rs7594951 loci of the *SRD5A2* gene in chronic HBV-infected patients. ns, *p* > 0.05; *, *p* < 0.05; **, *p* < 0.01

The relationship between *SRD5A2* rs12470143 and rs7594951 and serum HBV DNA levels was analyzed in chronic HBV-infected patients and stratified by age and sex. As illustrated in Fig. 2A, serum HBV DNA levels were significantly lower in the female patients than male patients. Further age stratification revealed that the HBV DNA levels in women aged ≤ 50 years old were still lower than those in men of the same age, whereas the serum HBV DNA levels in women aged > 50 years old were higher than in women aged ≤ 50 years old, and were similar with those in men of the same age (Fig. 2A). Furthermore, serum HBV DNA levels were significantly higher in patients with the T/T genotype compared to those with the non-T/T genotype at the rs12470143 and rs7594951 loci of the *SRD5A2* gene (Fig. 2B).

### Association of SRD5A2 protein levels with the METAVIR inflammatory activity/fibrosis degree and its difference in the male and female patients

**Fig. 3:**
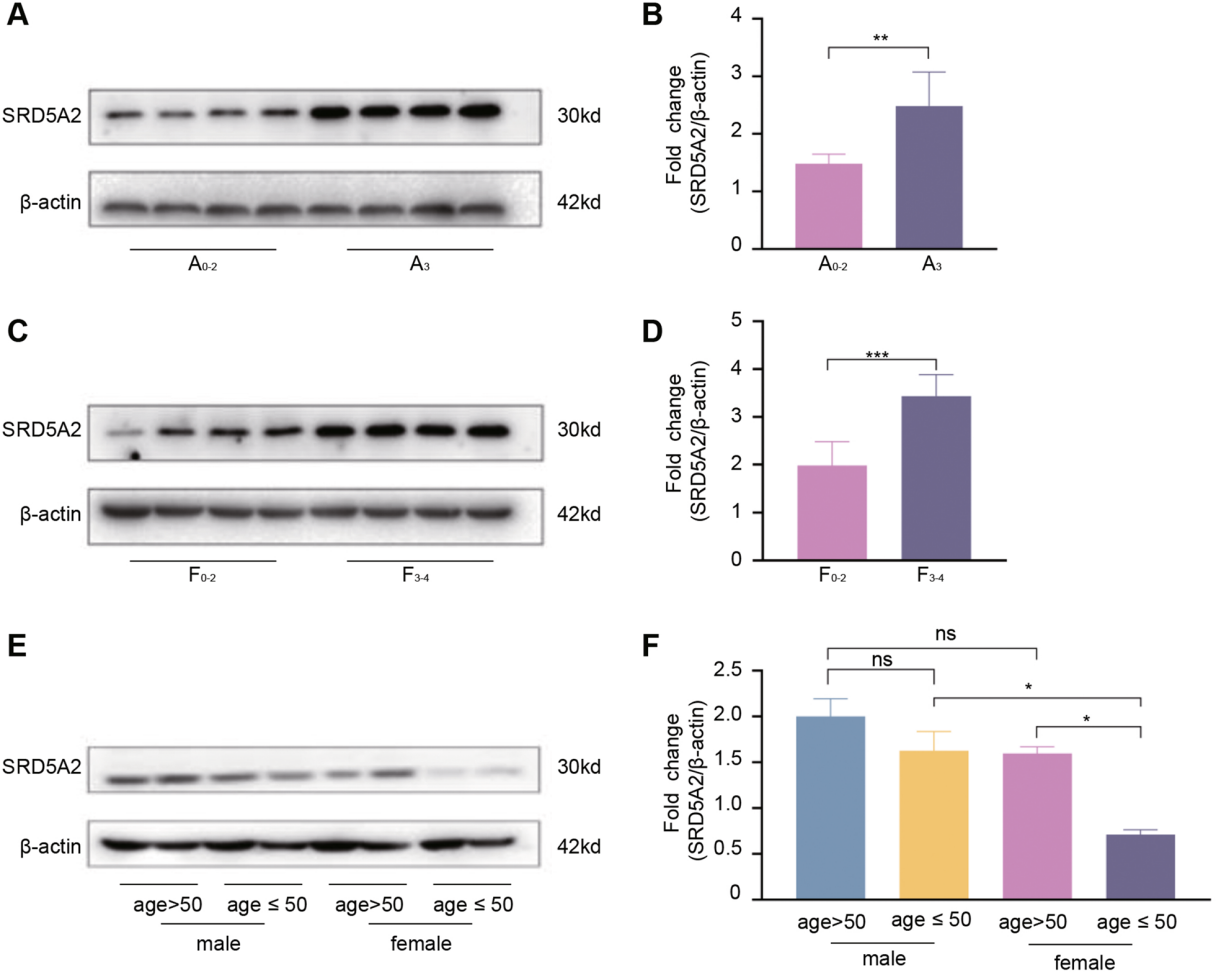
SRD5A2 protein expression levels in the liver tissues of chronic HBV-infected patients and their association with METAVIR inflammatory activity, fibrosis degree, and age. **A**, **B** SRD5A2 protein expression and its association with METAVIR inflammatory activity; **C**, **D** SRD5A2 protein expression and its association with the degree of liver fibrosis; **E**, **F** SRD5A2 protein expression levels among different age and sex groups

SRD5A2 protein expression was examined in 56 chronic HBV-infected patients with available percutaneous liver biopsy specimens, and its relationship with the METAVIR inflammatory activity/fibrosis degree was analyzed. As shown in Figs. 3A and B, SRD5A2 protein levels were significantly higher in patients with the METAVIR inflammatory activity score A3 compared with those with the score A0–A2 (*p* < 0.05). Similarly, SRD5A2 protein levels were significantly greater in patients with the METAVIR fibrosis score F3–F4 compared with those with the score F0–F2 (*p* < 0.05) (Figs. 3C, D).

After stratification by age and sex, SRD5A2 protein levels were analyzed and found to be significantly lower in female patients aged ≤ 50 years old than in male patients of the same age (*p* < 0.05), whereas they did not differ in female patients aged > 50 years old compared to in male patients of the same age (Figs. 3E, F). In addition, SRD5A2 protein levels did not differ between the two age groups of male patients, but differed significantly between the two age groups of female patients (*p* < 0.05) (Figs. 3E&F).

Among the 56 cases with chronic HBV infection who had undergone a liver biopsy, 53 cases were found to carry the non-T/T genotype of SRD5A2 rs12470143 and 3 cases the T/T genotype; all 56 cases were carriers of the non-T/T genotype of SRD5A2 rs7594951, with none carrying the T/T genotype. It is worth noting that the 3 cases with the T/T genotype of SRD5A2 rs12470143 were all male patients aged > 50 years old with METAVIR inflammation activity A3, and fibrosis degree F3–F4.

### Univariate and multivariate analyses of the risk factors for liver fibrosis in patients with chronic HBV infection

Univariate analysis showed that male sex, age > 50 years old, history of alcohol consumption, HOMA-IR > 2.5, HBeAg positivity, high HBsAg quantification, high serum HBV DNA load, viral genotype C, carrying the SRD5A2 rs12470143 locus C > T mutation, and carrying the SRD5A2 rs7594951 locus C > T mutation were risk factors significantly associated with the progression of liver fibrosis in patients with chronic HBV infection (all *p* < 0.05) (Table 5).

**Table 5:** Univariate and multivariate analyses of the risk factors associated with HBV-related liver fibrosis in patients with chronic HBV infection (n = 1007)

Variables	Univariate analysis		Multivariate analysis	
	OR (95%CI)	P	OR (95%CI)	P
Male	1.98 (1.50, 2.61)	** < 0.001**	1.84 (1.34, 2.54)	** < 0.001**
Age > 50 years old	3.41 (2.62, 4.45)	** < 0.001**	3.61 (2.69, 4.84)	** < 0.001**
Drinking	1.40 (1.07, 1.85)	**0.016**	0.95 (0.68, 1.31)	0.735
HOMA-IR ≥ 2.5	1.34 (1.03, 1.74)	**0.027**	1.35 (1.00, 1.82)	0.05
HBeAg positive	2.27 (1.58, 3.26)	** < 0.001**	2.14 (1.43, 3.21)	** < 0.001**
HBsAg load, log IU/mL	6.20 (3.49, 11.02)	** < 0.001**	6.32 (3.37, 11.88)	** < 0.001**
HBV load, log IU/mL	1.35 (1.24, 1.45)	** < 0.001**	1.34 (1.23, 1.46)	** < 0.001**
HBV genotype C	1.63 (1.17, 2.25)	**0.003**	1.63 (1.13, 2.35)	**0.008**
*SRD5A2 rs12470143* C > T	1.88 (1.15, 3.06)	**0.011**	1.79 (1.05, 3.05)	**0.033**
*SRD5A2 rs7594951* C > T	3.36 (1.73, 6.53)	** < 0.001**	4.55 (2.17, 9.54)	** < 0.001**

Further multivariate analysis identified a number of independent risk factors for HBV-related liver fibrosis, including male sex, age > 50 years old, positive HBeAg status, high HBsAg quantification, elevated serum HBV DNA load, HBV genotype C, and carrying the C > T mutation at the SRD5A2 rs12470143 or rs7594951 loci (Table 5).

## Discussion

Significant differences in chronic HBV infection between males and females have long been acknowledged, yet the underlying mechanisms have remained unclear. In this study, we analyzed 30 loci in six crucial genes involved in the regulation of sex hormone metabolism and signaling, and investigated their potential relationship with sex differences in disease severity among patients with chronic HBV infection. The major novel findings can be summarized as follows: (1) Two SNP loci in the *SRD5A2* gene (rs12470143 and rs7594951) were identified as displaying significant differences in allele frequencies between male and female patients with chronic HBV infection. The proportion of T alleles at the rs12470143 and rs7594951 loci of the *SRD5A2* gene was found to be more prevalent in male patients compared to female patients; (2) Patients with the T/T genotype exhibited a significant association with a higher incidence and severity of HBV-related liver fibrosis, as well as greater HBV DNA levels in patients with chronic HBV infection; (3) Elevated levels of hepatic SRD5A2 protein were significantly correlated with more severe liver inflammation and fibrosis in patients with chronic HBV infection; (4) The two genetic variants in the *SRD5A2* gene (rs12470143 C > T, r7594951 C > T), along with male sex and age over 50 years old, were identified as independent risk factors for the development of liver fibrosis among patients with chronic HBV infection. Collectively, this study suggests that SRD5A2 rs12470143 C > T and r7594951 C > T are associated with sex differences in the clinicopathological characteristics of patients with chronic HBV infection. Furthermore, male patents aged > 50 years old are at a higher risk of developing HBV-related liver fibrosis.

Earlier studies have demonstrated that HBV carriers with elevated testosterone levels have a significantly increased risk of HCC (OR = 2.06; 95% CI = 1.14–3.70) [22, 23]. In another study, 150 asymptomatic HBsAg carriers were followed up for up to 11.3 years (mean 6.1 years), and the results showed that women had a mean annual surface antigen clearance rate of 1.907%, an e antigen seroconversion rate of 12.9%, and a liver cancer incidence rate of 0.67%, whereas men had an annual HBsAg clearance rate of only 0.4%, an e antigen seroconversion rate of only 7.71%, and an incidence of hepatocellular carcinoma as high as 1.33%, which was twice as high as that of the women [24]. Studies in HBV transgenic experimental animals further support this theory that serum HBsAg and HBV DNA levels are significantly higher in male mice than in female mice at different growth stages. DeLoia et al. [25] found that adult transgenic male mice exhibited higher serum HBsAg levels than females, and that circulating HBsAg levels decreased by an average of 55.8 ± 5.2% and 55.5 ± 5.2% two weeks after surgical debulking, but returned to pre-surgical levels after testosterone supplementation. However, similar results have not been clinically confirmed. Indeed, a study of the anti-androgen compound flutamide in 32 patients with HCC who had no indications for surgery did not observe complete or partial remission [26]. Another multicenter double-blind clinical study [27] also showed no significant remission rates between the study groups in 244 patients with HCC who did not undergo surgery after being randomized in to groups with different anti-androgen or placebo regimens. There has long been considerable interest in the therapeutic effects of sex hormone intervention in chronic viral hepatitis, as well as in HCC. However, the discrepancies between animal experiments and clinical settings suggest that sex hormone levels alone cannot be used as a determinant, and that the role of sex hormone/sex hormone receptor-mediated signaling pathways and downstream molecules in the regulation of certain aspects may be relevant to the observed sex differences in HBV infection.

SRD5A belongs to a family of NADPH-dependent oxidoreductases, which consists of three family members (SRD5A1–3), among which SRD5A1 and SRD5A2 are primarily involved in steroid hormone metabolism and are predominantly expressed in the endoplasmic reticulum (ER) of human prostate cells and liver cells [28]. SRD5A1 [29] plays a role in generating 5-α-androstenedione from 4-androstene-3,17-dione as a substrate, and SRD5A2 [30] converts testosterone to dihydrotestosterone. Dihydrotestosterone exhibits a higher affinity for ARs compared to testosterone, resulting in a stronger biological activity. The 5-α-reductase encoded by *SRD5A2* is crucial in the conversion of testosterone to dihydrotestosterone, and its protein expression level or enzyme activity undoubtedly affects its reduction function as well as the synthesis/conversion of dihydrotestosterone [31, 32], which can bind to ARs, thereby activating androgen response elements in hepatocytes and promoting HBV mRNA transcription and disease progression. The findings of this study, which revealed significantly higher serum HBV DNA levels in HBV-infected patients with the T/T genotype compared to those with the non-T/T genotype at the rs12470143 and rs7594951 loci of the *SRD5A2* gene, further support the notion that SRD5A2 promotes androgen-regulated HBV DNA replication and disease progression, and its expression type correlates with these outcomes.

In chronic HBV-infected patients, the expression level of SRD5A2 may influence disease progression through its effects on the level and bioavailability of dihydrotestosterone. Additionally, it has been suggested [33] that knockdown of the *SRD5A2* gene can affect the binding activity of androgens to ARs, and their subsequent binding to the ARE, resulting in a downregulation of the AR downstream target gene TMPRESS2. Therefore, considering the findings of this study, it is reasonable to speculate that SRD5A2 expressed at the rs12470143 and rs7594951 loci with a T/T genotype may enhance the binding activity of dihydrotestosterone to ARs, promoting its binding to ARE on the HBV enhancer I subunit, thereby affecting HBV mRNA transcription and ultimately the progression of liver disease in chronic HBV-infected patients (Additional file 1: Fig. S1). It has been also demonstrated [34] that prostate tumor cells with increased levels of SRD5A2 expression exhibit significantly reduced matrix metallopeptidase 2 (MMT2) activity compared to control cells. Another study on the treatment of chronic liver disease indicated that MMP-2 plays a role in reversing the development of hepatocyte fibrosis by degrading various protein substrates in the extracellular matrix, including collagen and elastin [35]. In this study, male chronic HBV-infected patients with hepatic fibrosis at stage F4 had the T/T genotype at both the rs12470143 and rs7594951 loci of the *SRD5A2* gene, and hepatic SRD5A2 protein expression was significantly elevated. Furthermore, the severity of hepatic fibrosis was positively correlated with the level of SRD5A2 expression, suggesting that an elevated level of SRD5A2 expression may promote the development of fibrosis by reducing MMP-2 activity and impairing the degradation of extrahepatic matrix proteins. It is of note in this study that the expression level of SRD5A2 was significantly higher in female patients aged > 50 years old compared with those aged ≤ 50 years old, while the degree of liver fibrosis was significantly more severe in the former group. This suggests that, in addition to reduced matrix protein degradation, the high level of estrogen binding to HNF4α in reproductive women may also play a role, whereby it prevents HNF4α from binding to viral enhancer I, leading to decreased activity and the inhibition of HBV mRNA transcription.

As for the mechanism by which SRD5A2 polymorphisms affect the expression level of SRD5A2, it has been demonstrated [36] that the 3'-UTR site of SRD5A2 rs9332975 located in the exon 5 region is a target site for microRNAs. Subsequent microRNA binding to the target region may lead to translational repression or degradation of the target region and gene silencing, for which it is hypothesized that the C allele of rs9332975 in the 3'-UTR region may have a higher affinity for microRNA, leading to a downregulation of SRD5A2 gene expression. Additionally, Calais et al. [37] suggested that the c.544 G > A mutation in the *SRD5A2* gene may potentially disrupt the binding of U1snRNP to the 5 'splice site, thereby inhibiting splicing initiation and consequently SRD5A2 gene expression. Furthermore, it has also been observed [28] that mutations in certain sites of SRD5A2 can affect its 5α-reductase activity. In the *SRD5A2* gene, the most common mutation is located in exon 1 (p.Val89Leu), where the substitution of valine for leucine at position 89 was found to result in a 30% decrease in 5α-reductase activity [38]. Another mutation involves the change of codon 49 (p.Arg49Thr) from alanine to threonine, leading to a fivefold increase in 5α-reductase activity [39, 40]. The specific mechanism underlying these changes in activity may be related to alterations in the enzyme structure due to the amino acid substitutions. Moreover, mutations in SRD5A2 may impact the affinity of the substrate for NADPH and the binding domain of the ligand [28, 41], For example, the p.HisR171Ser mutation was revealed to markedly reduce the affinity of the enzyme for NADPH.

It is noteworthy that, to date, the generally higher SRD5A2 expression levels in men compared to women found in this study have received little attention. Mutations at the rs12470143 and rs7594951 loci in the *SRD5A*2 gene may enhance the efficiency of testosterone reduction and lead to an increased production of active dihydrotestosterone by affecting the activity of 5-alpha reductase. Specifically, mutations at these loci increase the efficiency of testosterone reduction and promote the production of more active dihydrotestosterone. The expression levels of SRD5A2 in male chronic HBV-infected individuals with non-T/T genotypes at the rs12470143 and rs7594951 loci of the *SRD5A2* gene were significantly higher than those in females. This indicates that the disease is more likely to progress in these individuals.

### Perspectives and significance

In conclusion, our research team conducted an analysis of the distribution of 30 polymorphisms from 6 genes associated with the sex hormone metabolism pathways in a cohort of 1007 patients with chronic HBV infection in Northeastern China. We performed a comprehensive stratification based on the severity of inflammation and fibrosis in liver tissue, as well as on sex and age. Our findings revealed that the expression patterns of the SNPs at the rs12470143 and rs7594951 loci of the *SRD5A2* gene and their sex-specific differences were not only associated with the HBV replication characteristics and the degree of inflammation/fibrosis in liver tissue, but also emerged as crucial factors influencing the sex-related disparities in disease characteristics among the patients with chronic HBV infection.

### Supplementary Information

**Additional file 1: Fig. S1.** Schematic representation illustrating the role of the androgen metabolism/conversion regulatory genes and their regulation of HBV transcription. **Fig. S2.** Schematic of estrogen inhibiting HCV transmission and/or entry by downregulating functional receptors. **Table S1.** Genotype and allele frequencies of 23 SNP loci in six genes between male and female chronic HBV patients.

## Data Availability

The datasets generated and analyzed during the current study are available from the corresponding author upon reasonable request.

## Abbreviations

SRD5A2: Steroid 5-alpha-reductase type 2
SNPs: Single nucleotide polymorphisms
HBV: Hepatitis B virus
AKR1C2: Aldosterone reductase 1C2
AKR1C3: Aldosterone reductase 1C3
HSD17B6: 17β-Hydroxysteroid dehydrogenase 6
SRD5A1: Steroid 5-alpha-reductase type 1
ESR1: Estrogen receptor 1
DNA: Deoxyribonucleic acid
CHB: Chronic hepatitis B
HBsAg: Hepatitis B surface antigen
HCC: Hepatocellular carcinoma
ARs: Androgen receptors
ARE: Androgen response element
HNF4α: Hepatocyte nuclear factor 4α
HCV: Chronic hepatitis C virus
E2: Estrogen
LSMs: Liver stiffness measurements
PCR: Polymerase chain reaction
H&E: Hematoxylin-eosin
HWE: Hardy–Weinberg equilibrium test
SD: Standard deviation
BMI: Body mass index
HOMA-IR: Homeostasis model of assessment for insulin resistance index
ALT: Alanine aminotransferase
AST: Aspartate aminotransferase
ALB: Albumin
PLT: Platelet
ER: Endoplasmic reticulum
TMPRESS2: Transmembrane serine protease 2
MMT2: Matrix metallopeptidase 2
3'-UTR: 3′-Untranslated region
NADPH: Triphosphopyridine nucleotide
