# Supplementary material for: Two genetic variants in the SRD5A2 gene are found to be associated with sex differences in the disease characteristics of patients with chronic hepatitis B virus infection
Source: Biol Sex Differ. 2023 Oct 3;14:68. doi: 10.1186/s13293-023-00553-4 (PMC10546680; doi:10.1186/s13293-023-00553-4)
Supplement: Supplementary file 1 — Additional file 1: Fig. S1. Schematic representation illustrating the role of the androgen metabolism/conversion regulatory genes and their regulation of HBV transcription. Fig. S2. Schematic of estrogen inhibiting HCV transmission and/or entry by downregulating functional receptors. Table S1. Genotype and allele frequencies of 23 SNP loci in six genes between male and female chronic HBV patients. [file 13293_2023_553_MOESM1_ESM.docx]

**Additional Information**

**
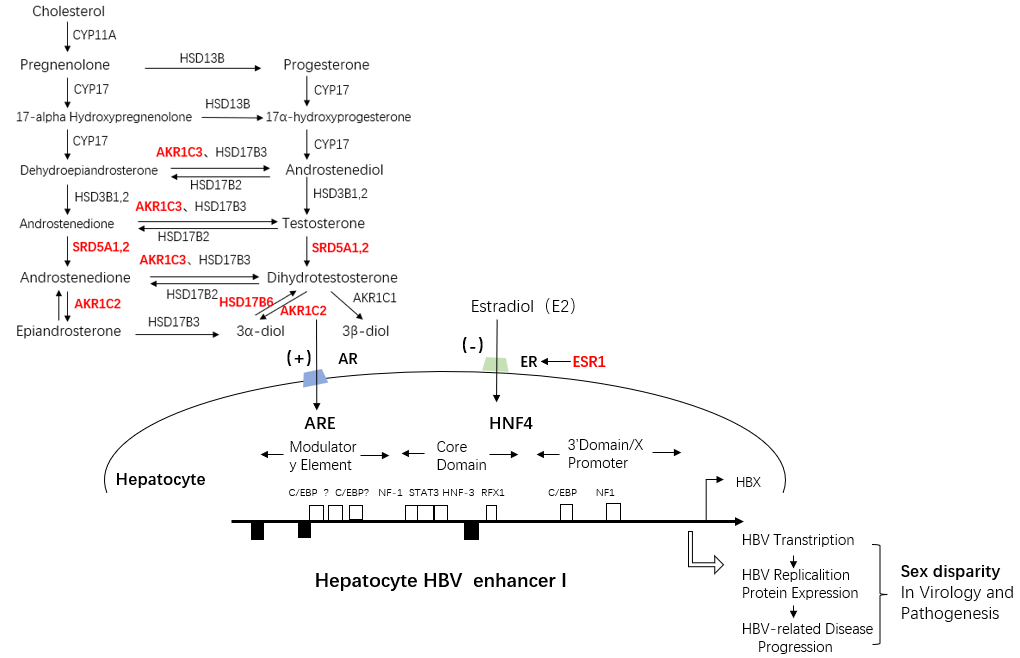
**

**Figure S1** **Schematic representation illustrating the role of the androgen metabolism/conversion regulatory genes and their regulation of HBV transcription.**


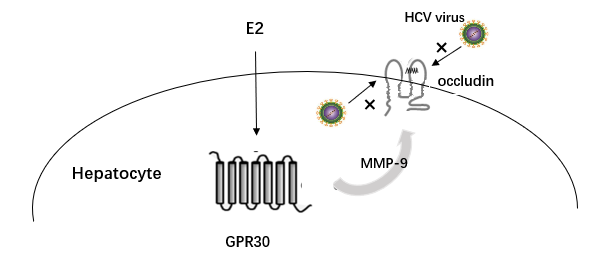


**Figure S2 Schematic of estrogen inhibiting HCV transmission and/or entry by downregulating functional receptors.**

**Table S1 Genotype and allele frequencies of 23 SNP loci in six genes between male and female chronic HBV patients**

| **Gene** | **SNP** **sites** | **Male (n=664)** | **Female (n=343)** | ***χ^2^*** | **P** |
| --- | --- | --- | --- | --- | --- |
| **AKR1C2** | **rs2801904** |  |  |  |  |
|  | C/C | 49 | 28 | 0.833 | 0.660 |
|  | C/A | 216 | 119 |  |  |
|  | A/A | 399 | 196 |  |  |
|  | C | 314 | 175 | 0.856 | 0.355 |
|  | A | 1014 | 511 |  |  |
|  | **rs12414884** |  |  |  |  |
|  | T/T | 457 | 225 | 1.181 | 0.554 |
|  | T/G | 173 | 97 |  |  |
|  | G/G | 34 | 21 |  |  |
|  | T | 1087 | 547 | 1.322 | 0.250 |
|  | G | 241 | 139 |  |  |
| **AKR1C3** | **rs2096421** |  |  |  |  |
|  | G/G | 44 | 26 | 1.200 | 0.549 |
|  | G/T | 225 | 125 |  |  |
|  | T/T | 395 | 192 |  |  |
|  | G | 313 | 177 | 1.225 | 0.268 |
|  | T | 1015 | 509 |  |  |
|  | **rs2398203** |  |  |  |  |
|  | C/C | 441 | 226 | 0.992 | 0.609 |
|  | C/T | 194 | 106 |  |  |
|  | T/T | 29 | 11 |  |  |
|  | C | 1076 | 558 | 0.030 | 0.863 |
|  | T | 252 | 128 |  |  |
|  | **rs2186174** |  |  |  |  |
|  | A/A | 441 | 226 | 0.992 | 0.609 |
|  | A/C | 194 | 106 |  |  |
|  | C/C | 29 | 11 |  |  |
|  | A | 1076 | 558 | 0.030 | 0.863 |
|  | C | 252 | 128 |  |  |
| **HSD17B6** | **rs4237805** |  |  |  |  |
|  | G/G | 486 | 266 | 4.884 | 0.087 |
|  | G/A | 161 | 64 |  |  |
|  | A/A | 17 | 13 |  |  |
|  | G | 1133 | 596 | 0.911 | 0.340 |
|  | A | 195 | 90 |  |  |
|  | **rs898611** |  |  |  |  |
|  | T/T | 19 | 13 | 0.772 | 0.680 |
|  | C/T | 219 | 116 |  |  |
|  | T/T | 426 | 214 |  |  |
|  | T | 257 | 142 | 0.528 | 0.467 |
|  | C | 1071 | 544 |  |  |
| **SRD5A1** | **rs3797177** |  |  |  |  |
|  | T/T | 392 | 215 | 1.663 | 0.435 |
|  | T/C | 240 | 110 |  |  |
|  | C/C | 32 | 18 |  |  |
|  | T | 1024 | 540 | 0.675 | 0.441 |
|  | C | 304 | 146 |  |  |
|  | **rs248800** |  |  |  |  |
|  | C/C | 412 | 195 | 1.888 | 0.389 |
|  | C/A | 215 | 85 |  |  |
|  | A/A | 37 | 13 |  |  |
|  | C | 1039 | 475 | 1.956 | 0.162 |
|  | A | 289 | 111 |  |  |
|  | **rs1691053** |  |  |  |  |
|  | T/T | 404 | 213 | 0.164 | 0.921 |
|  | T/C | 233 | 117 |  |  |
|  | C/C | 27 | 13 |  |  |
|  | T | 1041 | 543 | 0.158 | 0.691 |
|  | C | 287 | 143 |  |  |
| **SRD5A2** | **rs2208532** |  |  |  |  |
|  | G/G | 231 | 121 | 0.207 | 0.902 |
|  | G/A | 325 | 170 |  |  |
|  | A/A | 108 | 52 |  |  |
|  | G | 787 | 412 | 0.119 | 0.730 |
|  | A | 541 | 274 |  |  |
|  | **rs523349** |  |  |  |  |
|  | G/G | 212 | 110 | 0.935 | 0.626 |
|  | G/C | 338 | 182 |  |  |
|  | C/C | 114 | 51 |  |  |
|  | G | 762 | 402 | 0.276 | 0.599 |
|  | C | 566 | 284 |  |  |
|  | **rs4952197** |  |  |  |  |
|  | A/A | 126 | 71 | 1.214 | 0.545 |
|  | A/G | 328 | 157 |  |  |
|  | G/G | 210 | 115 |  |  |
|  | A | 580 | 299 | 0.001 | 0.970 |
|  | G | 748 | 387 |  |  |
|  | **rs612224** |  |  |  |  |
|  | C/C | 152 | 80 | 0.059 | 0.971 |
|  | C/A | 348 | 177 |  |  |
|  | A/A | 164 | 86 |  |  |
|  | C | 652 | 337 | 0.000 | 0.990 |
|  | A | 676 | 349 |  |  |
| **ESR1** | **rs6909023** |  |  |  |  |
|  | G/G | 488 | 255 | 2.618 | 0.270 |
|  | G/A | 167 | 88 |  |  |
|  | A/A | 9 | 1 |  |  |
|  | G | 1143 | 598 | 0.278 | 0.598 |
|  | A | 185 | 90 |  |  |
|  | **rs6920483** |  |  |  |  |
|  | G/G | 514 | 268 | 2.605 | 0.272 |
|  | G/A | 141 | 74 |  |  |
|  | A/A | 9 | 1 |  |  |
|  | G | 1169 | 610 | 0.351 | 0.554 |
|  | A | 159 | 76 |  |  |
|  | **rs2077647** |  |  |  |  |
|  | T/T | 262 | 135 | 4.197 | 0.123 |
|  | T/C | 326 | 153 |  |  |
|  | C/C | 76 | 54 |  |  |
|  | T | 850 | 423 | 0.910 | 0.340 |
|  | C | 478 | 261 |  |  |
|  | **rs2234693** |  |  |  |  |
|  | T/T | 248 | 129 | 1.822 | 0.402 |
|  | T/C | 327 | 158 |  |  |
|  | C/C | 89 | 56 |  |  |
|  | T | 823 | 416 | 0.339 | 0.561 |
|  | C | 505 | 270 |  |  |
|  | **rs1062577** |  |  |  |  |
|  | T/T | 315 | 167 | 1.791 | 0.408 |
|  | T/A | 288 | 137 |  |  |
|  | A/A | 61 | 39 |  |  |
|  | T | 918 | 471 | 0.046 | 0.830 |
|  | A | 410 | 215 |  |  |
|  | **rs7753153** |  |  |  |  |
|  | G/G | 368 | 194 | 1.065 | 0.587 |
|  | G/A | 269 | 131 |  |  |
|  | A/A | 27 | 18 |  |  |
|  | G | 1005 | 419 | 3.723 | 0.054 |
|  | A | 323 | 167 |  |  |
|  | **rs9340799** |  |  |  |  |
|  | A/A | 428 | 214 | 2.443 | 0.295 |
|  | A/G | 211 | 115 |  |  |
|  | G/G | 25 | 20 |  |  |
|  | A | 1067 | 543 | 1.827 | 0.176 |
|  | G | 261 | 155 |  |  |
